# A pruritic annular and polycyclic eruption

**DOI:** 10.1016/j.jdcr.2025.09.021

**Published:** 2025-10-02

**Authors:** Beatrix B. Thompson, Sushila A. Toulmin, Christopher Iriarte

**Affiliations:** a Harvard Medical School Boston, Massachusetts; b Department of Dermatology, Beth Israel Deaconess Medical Center, Boston, Massachusetts; c Harvard Combined Dermatology Residency Program, Boston, Massachusetts

**Keywords:** annular dermatoses, eosinophilic annular erythema, eosinophilic dermatoses

## Case description

**Fig 1:**
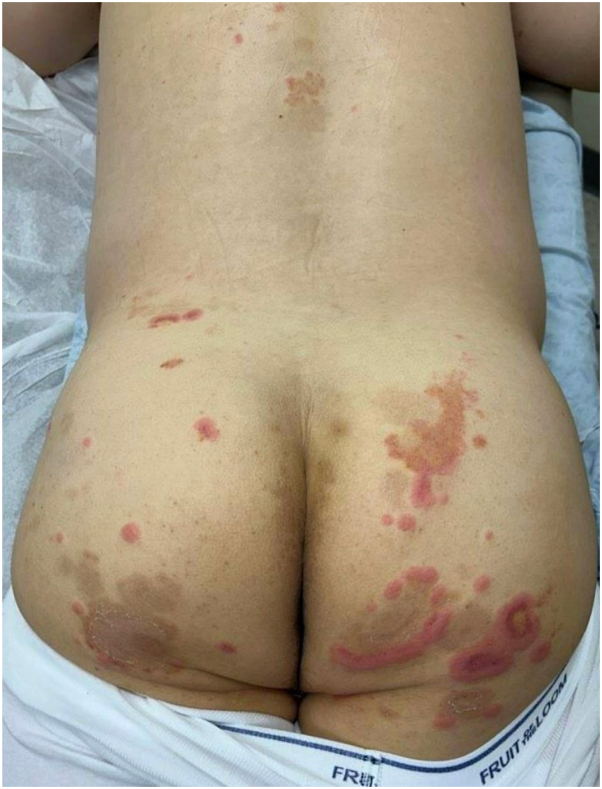
Buttocks and back at initial presentation.

**Fig 2:**
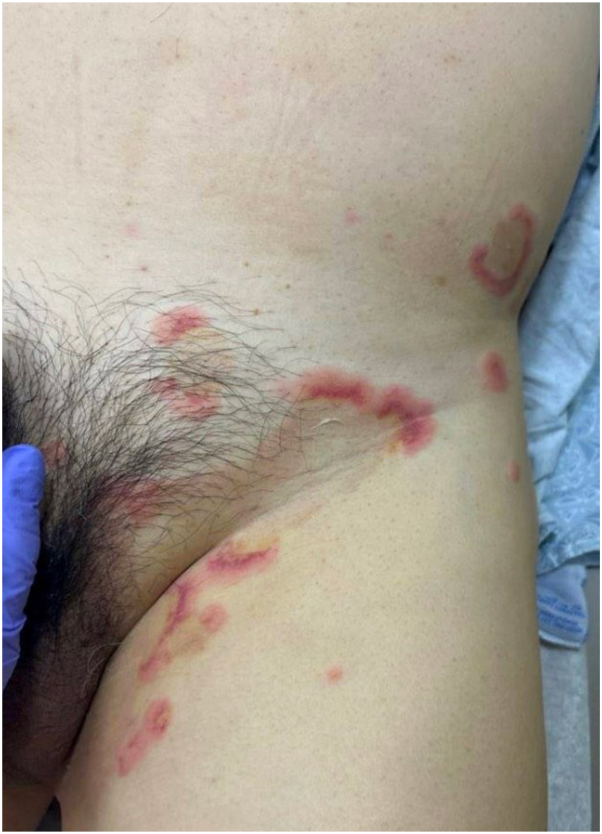
Left inguinal fold at initial presentation.

A 57-year-old male presented with 3 weeks of an intensely pruritic rash without associated systemic symptoms or preceding illness. He denied new medication exposures, apart from mushroom extract and taurine supplements he started several weeks prior. He had no improvement with topical or oral antifungals or topical corticosteroids. Physical examination demonstrated annular and serpiginous polycyclic plaques with an urticarial outer rim, violaceous inner border, and central golden hyperpigmented patches, over the mons pubis, buttocks, axillae, and inguinal folds (Figs 1 and 2). His dermatologic history was notable for 30 years of pruritic rashes that typically resolved within 24 hours, diagnosed as chronic idiopathic urticaria and managed with cetirizine.

**Fig 3:**
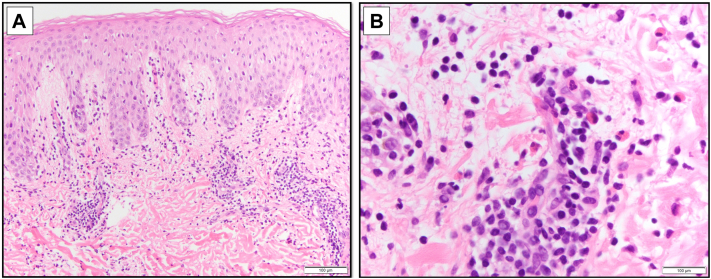
Hematoxylin and eosin-stained sections from skin biopsy of right buttock at **(A)** 10× and **(B)** 20× magnification.

He was advised to stop taking his supplements. Histopathology showed focal spongiosis, papillary dermal edema, and superficial and mid-dermal perivascular, periadnexal, and interstitial lymphocytic inflammation with prominent eosinophils (Fig 3), consistent with a diagnosis of eosinophilic annular erythema (EAE). Direct immunofluorescence studies and fungal cultures were negative. Laboratory evaluation was notable for hemoglobin A1c of 5.9%, negative human immunodeficiency virus and hepatitis C viral serologies, and normal mushroom immunoglobulin E. C-reactive protein, antinuclear antibodies, complements, rheumatoid factor, cryoglobulins, thyroid-stimulating hormone, complete blood count with differential, liver function tests, basic metabolic panel, urinalysis, and prostate-specific antigen were normal.

Following diagnosis of EAE, he started hydroxychloroquine 400 milligrams daily and a 4-week prednisone taper and resumed supplement use. He had complete clearance after 3 weeks of treatment. At this time, he self-discontinued therapy, and several days later, his rash recurred. He then resumed daily hydroxychloroquine. He has continued hydroxychloroquine monotherapy for 8 months with sustained clinical remission of skin disease.

**Question: Which of the following is most strongly associated with this cutaneous finding?**

A. Low serum complements
B. Immunosuppression
C. Monoclonal gammopathy
D. Recent group A Strep infection
E. Hematologic or solid organ malignancy

**Correct answer: E.**

## Discussion section

EAE is a rare annular eosinophilic dermatosis, characterized histologically by a dense perivascular and interstitial lymphocytic and eosinophilic infiltrate and basal melanosis.^1^ While the underlying etiology is unknown, EAE is hypothesized to result from a hypersensitivity reaction to an unknown antigen.^2^ The clinical differential diagnosis for EAE includes urticarial vasculitis, tinea corporis, subacute cutaneous lupus, and other annular erythemas. His rash lacked the classic flagellate morphology of mushroom dermatitis.

EAE has been associated with systemic triggers, including hematologic and solid tumor malignancies, hepatitis C, diabetes mellitus, renal disease, pancreatitis, hepatitis, eosinophilic granulomatosis with polyangiitis, and other autoimmune conditions.^1^^,^^2^ Our patient’s workup for underlying systemic causes was unrevealing. While many cases of EAE are idiopathic, he noted a temporal association of the rash with oral mushroom and taurine supplements, raising the possibility of supplement-induced EAE. However, he has sustained disease control on hydroxychloroquine monotherapy while taking these supplements, and thus it is unlikely that they are a causative agent of his eruption. To our knowledge, there are no prior definitive reports of supplement or medication-associated EAE.

There are no randomized controlled trials to guide EAE treatment. Prednisone with hydroxychloroquine has demonstrated the most consistent benefit in the literature.^2^ Recent case reports detail successful treatment with agents targeting interleukin-4 and interleukin-5.^2^, ^3^, ^4^ While rare reports detail spontaneous remission,^5^ EAE is overall considered a chronic dermatosis requiring maintenance therapy.

## Conflicts of interest

None disclosed.
